# Complete Sequences of the Mitochondrial DNA of the Wild *Gracilariopsis lemaneiformis* and Two Mutagenic Cultivated Breeds (Gracilariaceae, Rhodophyta)

**DOI:** 10.1371/journal.pone.0040241

**Published:** 2012-06-29

**Authors:** Lei Zhang, Xumin Wang, Hao Qian, Shan Chi, Cui Liu, Tao Liu

**Affiliations:** 1 College of Marine Life Sciences, Ocean University of China, Qingdao, Shandong, People’s Republic of China; 2 Beijing Institute of Genomics, Chinese Academy of Sciences, Beijing, People’s Republic of China; Leuven University, Belgium

## Abstract

The complete mitochondrial DNA (mtDNA) of *Gracilariopsis lemaneiformis* was sequenced (25883 bp) and mapped to a circular model. The A+T composition was 72.5%. Forty six genes and two potentially functional open reading frames were identified. They include 24 protein-coding genes, 2 rRNA genes, 20 tRNA genes and 2 ORFs (*orf60, orf142*). There is considerable sequence synteny across the five red algal mtDNAs falling into Florideophyceae including *Gr. lemaneiformis* in this study and previously sequenced species. A long stem-loop and a hairpin structure were identified in intergenic regions of mt genome of *Gr. lemaneiformis*, which are believed to be involved with transcription and replication. In addition, the mtDNAs of two mutagenic cultivated breeds (“981” and “07-2”) were also sequenced. Compared with the mtDNA of wild *Gr. lemaneiformis*, the genome size and gene length and order of three strains were completely identical except nine base mutations including eight in the protein-coding genes and one in the tRNA gene. None of the base mutations caused frameshift or a premature stop codon in the mtDNA genes. Phylogenetic analyses based on mitochondrial protein-coding genes and rRNA genes demonstrated *Gracilariopsis andersonii* had closer phylogenetic relationship with its parasite *Gracilariophila oryzoides* than *Gracilariopsis lemaneiformis* which was from the same genus of *Gracilariopsis*.

## Introduction

*Gracilariopsis* is an important economical marine red algae and widely used in food industry, agar extraction, etc [1], [2]. *Gracilariopsis lemaneiformis* has been cultivated on large scales in both the southern and the northern parts of China for food and agar production, playing an effective role against coastal eutrophication [3]. For the past few years, *Gracilariopsis* cultivation has become the third largest seaweed cultivation industry only after *Laminaria* and *Porphyra* in China. Among *Gracilariopsis* seaweeds, the *Gr. lemaneiformis* is one of the most important cultivated breeds, because of its high yields and commercially valuable extracts.

Mutation is one of the important means of breeding new varieties of *Gr. lemaneiformis*. Chemical mutagen treatments (EMS: ethyl methanesulphonate and N-methyl-N-nitrosoguanidine) could increase the frequency of mutations in *Gracilaria* species [4]. In China, two important commercial cultivated breeds of *Gr. lemaneiformis* (“981” and “07-2”) are both mutant stains. The strain “981” was bred from the wild *Gr. lemaneiformis* after mutation by N-methyl-N′-nitro-N-ni-trosoguanidine (MNNG) and selected with hydroxyproline (HYP) protocol. The strain “981” has the characteristics of high temperature tolerance, fast growth rate and high levels of content and quality of agar [5]. “981” has been extensively cultivated as a source of commercial agar in the coastal areas of Fujian and Guangdong Province of China. The strain “07-2” was obtained from the original “981” by the use of MNNG and selected with HYP protocol. The new strain (“07-2″) has excellent features of fast growing and strong resistance to bad environment [6].

Mitochondria are thought to be derived from eubacterial endosymbionts [7], because they have biochemical machineries to replicate and transcribe of their own genomes. Mitochondrial DNA has the features of compact size, mostly maternal inheritance and fast evolutionary rate, it is a good molecular marker for evolutionary and population studies [8], [9]. The *cox1* and *cox2-3* genes were good mtDNA taxonomic barcodes often used for species identification [10]. The whole mitochondrial genome makes it possible for the combination of multiple protein-coding genes, providing strong and precise phylogenetic analysis. However, limited information of complete mt genome is available. Since the first report on the complete mt genome of *Chondrus crispus* [11], there are only 6 red algal complete mt genomes deposited in the GenBank [12]–[14]. But for the seaweeds of brown algae (Phaeophyceae) and green algae (Chlorophyta), the complete mitochondrial data has reached 21 and 14 species respectively, more than that of red algal species. Here, we sequenced the complete mitochondrial genomes of wild *Gr. lemaneiformis* and two mutation strains. The comparison of the genome sequence and architecture reveals the influence of mutation to mtDNA. This work will contribute to further study about molecular phylogenetic analysis of red algae and germplasm improvement for the economic *Gracilariopsis* species.

## Results

### Genome Organization and Comparison

**Figure 1:**
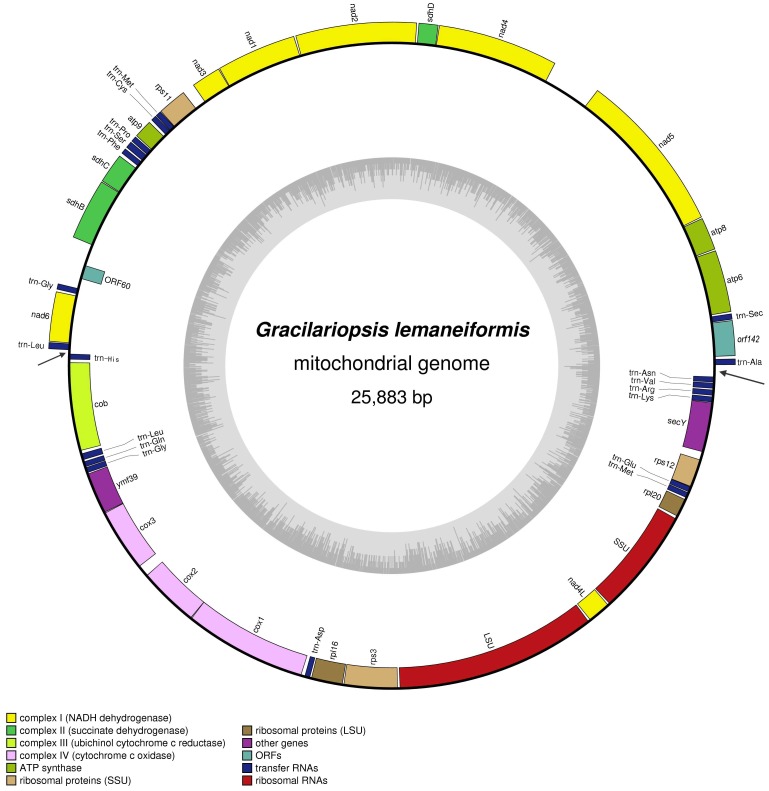
Gene map of *Gracilariopsis lemaneiformis* mitochondrial genome. Genes outside the map are transcribed counter clockwise and those inside the map are transcribed clockwise. Genes belonging to different functional groups are color coded. The black arrows indicate the positions of stem-loop and hairpin structures.

The mitochondrial DNA of *Gr. lemaneiformis* is a circular molecule of 25883 nucleotides (GenBank accession number: JQ071938), with an overall A+T content of 72.5%. This base composition is consistent with previously reports of red algal mitochondrial genomes [11]–[14]. Forty six genes including 24 protein-coding genes, 2 ribosomal RNA (rRNA) genes and 20 transfer RNA (tRNA) genes were identified. No intron is inserted in any of these genes and all genes are encoded on both heavy strand (H-strand) and light strand (L-strand) with approximately the same encoding proportion on each strand. The genes are encoded in two opposite major transcriptional directions, suggesting the existence of two main transcriptional units (Figure 1). Compared to the six known red algal mitochondrial genomes, the protein-coding gene content and order in all seven mt genomes are virtually identical. Besides, there is considerable sequence synteny across the five mitochondrial genomes which fall in Florideophyceae including *Gr. lemaneiformis* in this study and previously sequenced: *Gracilariopsis andersonii*, *Gracilariophila oryzoides*, *Chondrus crispus* and *Plocamiocolax pulvinata* [14] (Table 1).

**Table 1:** The comparisons of protein-coding genes and rRNA genes of five mitochondrial genomes from Florideophyceae.

Genes	*Chondrus crispus*	*Plocamiocolax pulvinata*	*Gracilariopsis andersonii*	*Gracilariophila oryzoides*	*Gracilariopsis lemaneiformis*
Protein-coding genes					
*atp6*	+	+	+	+	+
*atp8*	+	−	+	−	+
*atp9*	+	+	+	+	+
*cob*	+	+	+	+	+
*cox1*	+	+	+	+	+
*cox2*	+	+	+	+	+
*cox3*	+	+	+	+	+
*nad1*	+	+	+	+	+
*nad2*	+	+	+	+	+
*nad3*	+	+	+	+	+
*nad4*	+	+	+	+	+
*nad4L*	+	+	+	+	+
*nad5*	+	+	+	+	+
*nad6*	+	+	+	+	+
*sdhB*	+	+	+	+	+
*sdhC*	+	+	+	−	+
*sdhD*	+	+	+	+	+
*secY*	+	+	+	+	+
*ymf39*	+	+	−	+	+
*rpl16*	+	+	+	+	+
*rpl20*	+	+	+	+	+
*rps3*	+	+	+	+	+
*rps11*	+	+	+	+	+
*rps12*	+	+	−	+	+
Ribosomal RNA genes					
rRNA SSU	+	+	+	+	+
rRNA LSU	+	+	+	+	+

Compared to the mt genome of *Gr. andersonii* that from the same genus of *Gracilariopsis*, both genomes code for 26S rRNA gene (LSU) and 16S rRNA gene (SSU). The protein-coding gene content and order are almost identical with the following exceptions: the *ymf39* gene is present in the *Gr. lemaneiformis* genome between *cox3* and *cob* gene; a fragment of nearly 2000 bp contains three predict ORFs are characteristic in intergenic region of *Gr. andersonii* mt genome; the gene *rps11* appears in both mt genomes but in opposite directions; the gene length of *rps12* in *Gr. andersonii* is 129 bp shorter than that of *Gr. lemaneiformis* for a premature stop codon. In addition, in mt genome of *Gr. lemaneiformis,* a fragment of ∼600 bp containing a predicted ORF (*orf60*) is inserted into intergenic region between *sdhB* and *nad6* gene; another fragment of ∼150 bp containing a stem-loop structure is present between *atp6* and *secY* gene (trn-Ala and trn-Asn, Figure 1).

### Gene Content and Codon Usage

The mtDNA of *Gr. lemaneiformis* codes for three subunits of the cytochrome oxidase (*cox1-3*), apocytochrome b (*cob*), seven subunits of the NADH dehydrogenase complex (*nad1-6, nad4L*), four ATPase subunits (*atp6*, *atp8*, *atp9*, *ymf39*), five ribosomal proteins (*rps3, rps11, rps12, rpl16, rpl20*) and three succinate dehydrogenase complex (*sdhB, sdhC*, *sdhD*). Start and stop codons of all protein-coding genes were determined based on alignments with the corresponding genes and proteins of other Florideophyceae species. As shown in Table 2, the codon ATG was used as start codon for all genes, the stop codons include two types: TAA and TAG.

**Table 2:** Characteristics of the mitochondrial protein-coding genes of *Gracilariopsis lemaneiformis*.

		Codon			
Gene	Size (bp)	Amino acid	Start	Stop	Strand
*apt6*	762	253	ATG	TAA	H
*atp8*	405	134	ATG	TAA	H
*nad5*	1992	663	ATG	TAA	H
*nad4*	1476	491	ATG	TAG	H
*sdhD*	240	79	ATG	TAA	H
*nad2*	1476	491	ATG	TAA	H
*nad1*	981	326	ATG	TAA	H
*nad3*	366	121	ATG	TAA	H
*rps11*	360	119	ATG	TAA	H
*atp9*	231	76	ATG	TAA	H
*sdhC*	372	123	ATG	TAA	H
*sdhB*	753	250	ATG	TAA	H
*nad6*	609	202	ATG	TAG	H
*cob*	1143	380	ATG	TAA	L
*ymf-39*	543	180	ATG	TAA	L
*cox3*	819	272	ATG	TAA	L
*cox2*	792	263	ATG	TAA	L
*cox1*	1596	531	ATG	TAA	L
*rpl16*	411	136	ATG	TAA	L
*rps3*	696	231	ATG	TAA	L
*nad4L*	306	101	ATG	TAA	L
*rpl20*	243	80	ATG	TAG	L
*rps12*	369	122	ATG	TAG	L
*secY*	735	244	ATG	TAA	L

Twenty tRNA genes were determined and show standard cloverleaf secondary structures with the length ranging from 70–88 bp. All tRNA genes are dispersed throughout the *Gr. lemaneiformis* on both strands (Figure 1). Like all the other algal mt genomes published, the genome did not contain all the tRNAs needed to complete translation alone.

### Secondary Structures

**Figure 2:**
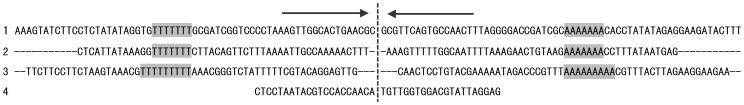
The comparison of sequences of stem-loop and hairpin structure from different mt genomes. **1**: *Gracilariopsis lemaneiformis* (126 bp). **2**: *Plocamiocolax pulvinata* (102 bp). **3**: *Chondrus crispus* (116 bp). **4:** the hairpin structure of *Gr. lemaneiformis* (42 bp). The dotted line indicates the middle point from where both sides are complete inverted repeat sequences. The shadow indicates polymers of A and T (n = 6, 8).

At the demarcation point of the two opposite transcriptional units, a long and stable stem-loop (63 nt) was identified in intergenic region of mt genome of *Gr. lemaneiformis*. Nearly diametrally opposite on the circular mt genome, another transcriptional demarcation point between trn-Leu and trn-His (Figure 1), a hairpin structure (21 nt) was found. Both secondary structures were complete inverted repeat sequences. The stem-loop and hairpin structures are likely to cause the termination of transcription, moreover, inverted repeat sequences provide the same binding site of enzyme at different directions. So, these structures could be involved in DNA transcription. Such strict stem-loop structures are also present in intergenic regions of *Chondrus crispus* (58 nt) and *Plocamiocolax pulvinata* (51 nt) mt genomes [11], [14], and also locate at the demarcation point of different transcriptional units. There was no visible sequence homology between these secondary structures even with similar sequence length. However, all of these three inverted repeat sequences contain polymers of A and T (n = 6, 8, Figure 2). The polymers with low free energy tend to unlink easily and may be a recognition site of some enzymes. So the stem-loop structure is putative origin of replication of mtDNA.

### Comparison of mtDNA of Wild and Two Mutation Strains

The complete mt genomes of two mutation strains (“981”, “07-2”) were sequenced using the same method as the wild *Gr. lemaneiformis*. The mitochondrial genome size and gene order of the two mutation strains were the same as the wild *Gr. lemaneiformis*. Only nine base mutations were detected and all base mutations located at gene coding regions. Of which, eight were present in the protein-coding genes and one in the tRNA gene. The protein-coding genes contained base mutations were *atp6*, *nad5*, *rps11*, *sdhC* and *cob* genes. However, none of these base mutations caused frameshifts or a premature stop codon in the mtDNA genes. The only one mutation present in tRNA gene did not make any change to the structure and function of *tRNA^Pro^*. Among the eight base mutations present in the protein-coding genes, five of them are samesense mutations and the other three are nonsynonymous mutations (Table 3).

**Table 3:** Comparison of mtDNA base mutations between wild *Gr. lemaneiformis* and two mutation strains.

Gene	Position	wild	“981”	“07-2″	Strand
*apt6*	732	AT**T** (Ile)	AT**T** (Ile)	AT**C** (Ile)	H
*nad5*	1092	CA**G** (Gln)	CA**A** (Gln)	CA**G** (Gln)	H
*rps11*	188	T**G**T (Cys)	T**G**T (Cys)	T**A**T (Tyr)	H
*tRNA^Pro^*	27	**A** (trn-Pro)	**G** (trn-Pro)	**A** (trn-Pro)	H
*sdhC*	93	TT**C** (Phe)	TT**C** (Phe)	TT**T** (Phe)	H
*cob*	756	CA**T** (His)	CA**T** (His)	CA**C** (His)	L
*cob*	30	TC**T** (Ser)	TC**C** (Ser)	TC**T** (Ser)	L
*cob*	25–26	**CT**T (Leu)	**AC**T (Thr)	**CT**T (Leu)	L

### Phylogenetic Analyses

**Figure 3:**
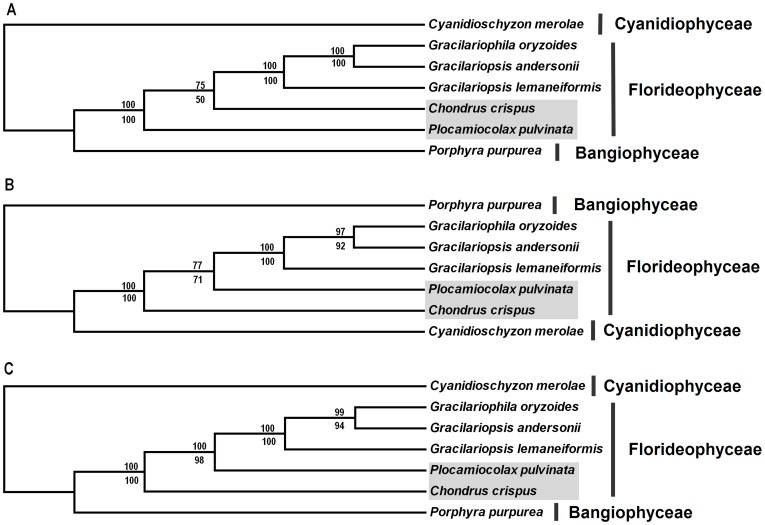
ML and MP trees of seven red algae based on different data partitions of mtDNA. **A**: 19 protein-coding genes. **B**: LSU. **C**: SSU. The ML and MP trees have the same topology and only one is shown. Numbers above and below nodes are ML and MP bootstrap support values respectively. The shadow indicates topological difference between trees based on different data partitions.

Three data partitions (19 protein-coding genes, LSU and SSU) from seven red algal mt genomes were used to construct phylogenetic trees. The aligned sequence length of protein-coding genes, LSU and SSU were 15915 bp, 2882 bp and 1573 bp respectively. Phylogenetic trees with bootstrap values are present in Figure 3. Both MP and ML analyses yielded the same trees for each data partition and phylogenetic trees of three data partitions were similar with each other, in which *Cyanidioschyzon merolae* and *Porphyra purpurea* formed single clade as they belong to Cyanidiophyceae and Bangiophyceae respectively; the remaining five species falling into the same class (Florideophyceae) clustered together (Figure 3). Among three Gracilariaceae species, phylogenetic analyses demonstrated *Gr. andersonii* had closer relationship with *Gracilariophila oryzoides*, even though *Gr. andersonii* and *Gr. lemaneiformis* belong to the same genus of *Gracilariopsis*. The topological differences were mainly occurred within Florideophyceae clade. Within this clade, the branching order of *Plocamiocolax pulvinata* and *Chondrus crispus* were different among the phylogenetic trees based on different data partitions of mtDNA: *Plocamiocolax pulvinata* clustered out ahead of *Chondrus crispus* when using the data partition of protein-coding genes, whereas the branch order was opposite when using the data partition of LSU and SSU (Figure 3).

## Discussion

To date, the complete mitochondrial genomes of seven red algae (including this work) have been reported. According to the results of previous studies and our research, the mtDNA sizes of red algae are ranging from 25.1 kb to 36.7 kb, and the A+T content of these mitochondrial genomes are 72.0–76.1% [11]–[14]. However, for the seaweeds, the mtDNA sizes of brown and green algae are in the range of 31.6–58.5 kb and 12.9–95.8 kb respectively. It is obvious that there exists great variance between mt genomes of seaweeds, including the kinds of protein-coding genes, species of tRNAs and rRNA genes. Moreover, the mtDNAs of green algae have special model of linear molecule and circular molecule [15], [16]. In addition, the mtDNAs of brown and red algae were both circular molecule and encoded on H-strand and L-strand, but the encoding proportion of two strands are very different. For red algae, the encoding proportion of each strand is nearly the same; while the L-strand of brown algae just encodes several ribosomal protein genes, the other protein-coding genes and all tRNAs genes are encoded on H-strand, indicating a clear strand-specific bias in encoding genes. As noted by Gray, the mitochondrial genomes of red algae are incredibly well conserved in both content and genome architecture [17]. In despite of the discrepant mt genome size of red algae, the A+T content remains at a relatively stable level. Especially for the five species from Florideophyceae, the number and order of mtDNA genes are almost the same (Table 1).

According to previous reports, the mitochondrial genome size had undergone a substantial expansion from the green algae *Chara vulgaris* to land plants [18], which was mainly accounted for the enlargement of intergenic regions and duplications, and also get DNA from the chloroplast and the nucleus [19]–[22]. However, such phenomenon has not been observed in the mt genomes of brown and red seaweeds except for few species have few introns [14]. For the seven known mt genomes of red algae, compared to the primitive red alga *Cyanidioschyzon merolae* from Cyanidiophyceae, the genome sizes of other six species from Bangiophyceae and Florideophyceae show a process of decreasing [11]–[14], the mitochondrial genomes of red algae have a trend to become smaller and more stable.

### Codon Usage

For the 24 protein-coding genes of mt genome of *Gr. lemaneiformis*, there are just two types of stop codon, TAA and TAG. As reported before, special in the mitochondrial genome, the modified genetic code TGA specifies tryptophan instead of the stop codon [23]. These modifications are considered to be correlated to an A+T pressure [24]. Like the green-plant *Acanthamoeba castellanii* which displays a mitochrondrial A+T content of 71% use a modified genetic code, whereas another green alga *Prototheca wickerhamii* has the similar A+T content of 74% but with no usage of TGA as stop codon nor tryptophan [25], [26]. This situation of a deviant genetic code was observed in our research, and the codon TGA distributed over nearly all the genes of complete mt genome of *Gr. lemaneiformis*. For the known mt genomes of green and brown algae, we chose the gene *cox1* for a simple search, the results indicated no modified genetic code TGA was used in brown algae and only one was present in green algae. However, all the seven red algal mt genomes contain different numbers of modified genetic code TGA, it can be said this modified genetic code has the highest frequency in red algae. Moreover, such phenomenon was also observed in *cox1* genes of other red algal genera of *Eucheuma* and *Kappaphycus* [10].

### Influence of Mutation

Colour mutants are relatively widespread in red algae [27]. Moreover, chemical mutagen treatments can increase the frequency of mutations [4]. For *Gr. lemaneiformis*, spontaneous colour mutants have been observed in the nature environment. Early in 2002, Sui found that the absorption spectra of phycoerythrins (PE) changed significantly between *Gracilaria lemaneiformis* Greville and its pigmental mutants [28]. The two mutants (“981” and “07-2″) used in this study were the products mutated by chemical mutagenesis and selected the strains with excellent characteristics. We sequenced the complete mt genomes of the two mutants, the comparison of mtDNA between the mutants and wild *Gr. lemaneiformis* revealed that there was no obvious variance except several amino acids changing caused by base mutations. Among the eight base mutations present in the protein-coding genes, five of them are samesense mutations and cause no impact to the gene structure and gene function. The other three are nonsynonymous mutations, but there is no significant clue for the influence on the protein-coding genes except an amino acid change. These results suggest that the mutation does not cause the large-scale rearrangements and obvious insertion or deletion to *Gr. lemaneiformis* mt genome. To further understand the exact gene in relevant to the new characteristics particular for mutants, more efforts need to be done.

### Origin of Replication

As reported before, the mitochondrial genome of mammalian has the displacement-loop (D-loop) which also known as the control region, including transcriptional promoters for both strands and the heavy strand replication origin [29], [30]. This noncoding region attracted many studies for its supposedly rapid rate of evolution [31]. In our research, a length of 63 nt complete inverted repeat sequence was detected in intergenic region of mt genome of *Gr. lemaneiformis*, and the stem-loop structure located at the demarcation point of two transcriptional units. This inverted repeat sequence seems like the D-loop structure of animal mt genome that can cause the termination of transcription, so it was presumed to be the origin of replication and in relevant with transcription. Similar structures were found in *C. crispus* and *P. pulvinata* mt genomes, and in *P. pulvinata* it was considered to be the origin of replication [14]. However, these strict secondary structures were only found in three mt genomes of red algae, and no similar sequence was found throughout the all known mt genomes of brown algae. Interestingly, even *Gr. andersonii* and *Gr. lemaneiformis* both belong to the genus of *Gracilaropsis*, no such structure was found in the mt genome of *Gr. andersonii*.

In our research, the complete mitochondrial sequence was obtained by PCR strategy and sequenced by dideoxy chain termination method, the instability of PCR reaction may cause the deletion or base mutation of DNA fragments, special for the region include secondary structure. Frequently, normal rTaq enzyme can easily cross the folded hairpin structure and result in the loss of partial PCR fragment. Therefore, the accuracy and integrity of sequence must be considered when choosing the strategy of PCR to obtain the complete mitochondrial genome.

## Materials and Methods

### Ethics Statement

As a normal kind of economical red algae, no specific permits are required for the studies on *Gr. lemaneiformis* so far. The wild *Gr. lemaneiformis* was collected from Shandong Province of China in May 2011, the two mutation strains (“981”, “07-2”) were collected from Fujian Province of China in April 2011. All materials were collected in the coastal area and the location is neither privately-owned nor protected places.

### DNA Isolation, Genome Sequencing

Total DNA was extracted from fresh thalli with modified CTAB method [32]. mtDNA of *Gr. lemaneiformis* was amplified by polymerase chain reaction (PCR) using primers (Table S1) designed in Primer Premier 5.0 from the conserved regions of the known red algal mitochondrial genomes [11]–[14]. The PCR reaction conditions include an initial denaturation cycle of 94°C for 3 min followed by 30 cycles of 94°C for 30 s, annealing temperature depending on primer sequences for 45 s, and 72°C for 1 min. A final extension step was performed at 72°C for 15 min followed by a 4°C hold. For large fragments, the extension time depend on the sequence length (approximately 1 min for 1,000 bp). PCR products were purified and sent for sequencing on an ABI 3730 automated sequencer (Applied Biosystems). Individual sequences were edited and assembled into contigs using DNAStar (DNASTAR, Inc., Madison, USA).

### Genome Annotation

Genes were identified using the BlastN and BlastX algorithms to compare the predicted ORFs to the NCBI GenBank database [33]. Ribosomal RNA (rRNA) genes and coding regions were determined by sequence alignment with the known mitochondrial genes of *Gr. andersonii* and *Gracilariophila oryzoides* [14]. Transfer RNA (tRNA) genes were identified with tRNAscan-SE 1.21 software [34]. Mitochondrial genome map of *Gr. lemaneiformis* was produced by using OGDRAW software [35]. Sequence alignment and base composition were conducted using MEGA 4.0 software [36].

### Phylogenetic Analyses

Phylogenetic analyses of the red algal species were performed based on the following data set: 19 protein-coding genes (*atp6*, *atp9*, *cob*, *cox1*, *cox2*, *cox3*, *nad1*, *nad2*, *nad3*, *nad4L*, *nad4*, *nad5*, *nad6*, *rpl16*, *rps11*, *rps12*, *rps3*, *sdhB*, *sdhD*), 26S rRNA gene (LSU) and 16S rRNA gene (SSU). All sequences are commonly present in all seven mt genomes publicly available in the GenBank database. Sequences were aligned using MEGA 4.0 [36] software and edited manually. Maximum parsimony (MP) analyses were performed with PAUP*4.0b10 [37]. Heuristic searches were conducted with 1000 random addition replicates and tree bisection reconnection (TBR) branch swapping with “multrees” option. For maximum likelihood (ML) analyses, RA×ML version 7.0 [38] was used with searches relied on the general time-reversible (GTR) and gamma models. The local bootstrap probability of each branch was calculated by 1000 replicates. MEGA 4.0 was used for displaying and printing phylogenetic trees.

## Supporting Information

Table S1 Primers sequences designed to sequence three strains of *Gracilariopsis lemaneiformis*.
(DOC)
