# Supplementary material for: Complete Sequences of the Mitochondrial DNA of the Wild Gracilariopsis lemaneiformis and Two Mutagenic Cultivated Breeds (Gracilariaceae, Rhodophyta)
Source: PLoS One. 2012 Jun 29;7(6):e40241. doi: 10.1371/journal.pone.0040241 (PMC3386957; doi:10.1371/journal.pone.0040241)
Supplement: Table S1 — Primers sequences designed to sequence three strains of Gracilariopsis lemaneiformis. (DOC) [file pone.0040241.s001.doc]

**Table S1:** Primers sequences designed to sequence three strains of *Gracilariopsis lemaneiformis*.

|  | Primer Sequence | |  |  |
| --- | --- | --- | --- | --- |
| Primer Name | Forward primer (5'-3') | Reverse primer (5'-3') | Product Size (bp) | Annealing Temperature (℃) |
| Ga-2 | GTTTTATACAATTTTAACCCAC | GGATAGACAATTATAGCATTTA | 318 | 44.2 |
| Ga-6 | CCGCTTGTAGGATTAGTA | ATCCATAACCACCCATTT | 736 | 44.7 |
| Ga-10 | GGCAAGTGTAAAGCGTAC | TACAGTTAAAGCGAAAGC | 715 | 45.4 |
| Ga-14 | ATGAATGTAACTTTACAAAGTG | AGCCATCATTAAAGCAAA | 210 | 45.9 |
| Ga-18 | AATACTTGCTATTTGACC | ATGAGGTGCTACTGTTAT | 649 | 44.3 |
| Ga-19 | TTCCAGATCCATCTTGAT | GTCCAATTATTTCCATAGTAAA | 603 | 45.7 |
| Ga-21 | AACAATGGGCATAAATCC | CAAGATCCAGCTACACCA | 633 | 46.7 |
| Ga-23 | AACGGCACCCATAGATAA | AAAGCCATTCAGGAGGAG | 749 | 48.2 |
| Ga-24 | TATAAACTTCAGGATGTC | CATAAAGATATTGGAACT | 706 | 44.4 |
| Ga-25 | CAAATTCAAATAGAACCATAC | GGAAAATAAAACGCATAA | 314 | 43.9 |
| Ga-28 | AATTGGGTGGTATTTCAT | TAAGTTAGTCGGGCCTTA | 699 | 45.8 |
| Ga-29 | TAATTTCCTATCGAGTAC | ACCTGAAACCAAGTGATC | 711 | 43.2 |
| Ga-30 | AAGTTTGATTAGTCTTTCGCTC | AATTTTGGACGTCTCGCT | 638 | 47.7 |
| Ga-32 | TTGTTACGACTTCACTTC | CTATAGTGTAAACGATAATCTA | 700 | 46.6 |
| Ga-33 | CCACATGCTCCACTGCTT | AATGGTGAACGGGTGAGT | 764 | 48.8 |
| Ga-41 | TGGGGTTTGCTCTTTTCT | ATGGGTTAAATCCACATTCATA | 472 | 46.0 |
| Ga-42 | AAGGAGCTTTAGAATGAG | TAACGGAAATAGGACTTG | 602 | 43.3 |
| Ga-46 | AATGCGTTTATTTAGATGTC | ACCTTTCCAACTGCTTGT | 635 | 45.4 |
| Ga-52 | TGTCATAATGTACGGCTTAA | AGCAGGAATGCCTAGAAG | 493 | 46.3 |
| Ga-53 | TCAAAGGAGGATAAACTG | AATGGGTAAAGGTAAAGG | 720 | 46.0 |
| Ga-55 | AAATCTCATACTTGGGTTAG | GGTACGTGAGTTGGGTTC | 394 | 45.0 |
| Ga-57 | TTAACCTGCTCACAACTA | GAAGTGTTATTACCGTAGA | 652 | 44.2 |
| Ga-60 | GCCCAAGATTCCTCACTG | AACGCAAAGTCGTAAACAAA | 569 | 46.9 |
| Bc-1 | TAGTACCCTTAACATTCTTTGG | AATAAACCGCCTGCTGAA | 571 | 46.9 |
| Bc-4 | TGAGAAGGTGTCGGTTTA | ACGACGCATATCTTGTTC | 687 | 47.0 |
| Bc-11 | CTGCGGTCCTATGGTATT | TTGCTTTTCCAGGATTTA | 578 | 46.7 |
| Bc-16 | CAGAAAAGATGCTGATAT | ATTAGGAGGTTGTATGTC | 630 | 45.2 |
| Bc-18 | ATCCACAGCTACCTAATT | GGTCTAACCCAAGTATGA | 628 | 43.5 |
| Bc-19 | TGCTACTGGCATAACAAT | TAAGACGGAAAGACCCTA | 647 | 46.8 |
| GJ1-1 | AACAGCCATACGCTCTAC | TTAAAAGCTCGCTATTCA | 652 | 45.2 |
| GJ2-1 | TAAGCGAAATAGGGGAATA | ATAAAGGAACAATAGGTAAAGGT | 590 | 46.6 |
| GJ3-1 | ACCTTTACCTATTGTTCCTTTAT | GTTTGCATCTATGATTTGTCTA | 465 | 45.8 |
| GJ4-1 | CAGTTGCTAAATGCTATA | GAATAATCAGATACTAAAGG | 687 | 43.7 |
| GJ5-1 | GCAGGTTCAGTAATACAC | ATAGGTCCAATACGCTAA | 1938 | 43.1 |
| GJ5-4 | ATTAGGGGTTTGTTTATTTG | GCGGGAGTAAGTGAAGTT | 741 | 44.8 |
| GJ6-3 | TAGGTTCCACGGATTTAC | CAGCTCCCATTACCATTC | 697 | 46.8 |
| GJ7-2 | TAACATAAAGGGATTGGC | ATGCGTACTTTGAATTACTA | 799 | 45.5 |
| GJ8-2 | TTTTAGGAGGTTGGTTAC | AAGGGATCATGTACTTAT | 664 | 42.9 |
| GJ9-2 | ACAACTTCTGGATTAAAACGAA | ACTCCCAAAGCAAACACG | 736 | 48.2 |
| GJ10-1-1 | GTTAGAGCATAGGGTTGA | GTCGGAAAGTTAAAGTAGA | 659 | 43.8 |
| GJ10-2-2 | AGAGGTTAGAGCATAGGGTTGA | CGTCGGAAAGTTAAAGTAGAAT | 688 | 46.1 |
| GJ11-1 | TTTACTCCAAGCGTATCGT | TGAGCCTACTGTTAATTCTGTG | 544 | 46.1 |
| GJ12-1 | AGCAGGTTTACTTTTACTTTTAGG | ATCTTGGGATGAATAGGTGGTATG | 744 | 47.3 |
| GJ13-1 | TTTATTGGTGTTGGATAA | CCTACAGTTGAAATTGGT | 1398 | 43.8 |
| GJ14-2 | AATAACATAACAAAACCG | GAAATAATATCACCAGCG | 767 | 44.5 |
| GJ15-1 | TATGACCCATACGACGCT | CCACTTTTTTTGACCCTG | 725 | 48.8 |
| GJ16-1 | AGCCAACAGTAACAGAAA | ATAAGCAATGGGAGAGAA | 740 | 46.6 |
| GJ17-1 | AAGATTTCTCATTTGCTG | AAGTCGTTTATTCGTCGA | 600 | 44.1 |
| GJ18-1 | TTTGCCAAGGTTCTCATT | GTTTTGTACACACCGCCC | 540 | 46.5 |
| GJ19-3 | TTTATACTCCTAGTGGGA | TTTTTGTAGAATTTCGTC | 758 | 44.2 |
| DL1-1 | ACGACCGCCATAAAACTA | GTGCTGAAAAAAACTCCA | 757 | 44.8 |
| DL2-1 | CGTTTTGGTTGTTTTTTA | GGCTTATTTTTTTCGTTA | 747 | 43.5 |
| DL3-1 | CTTGTAATGTATGTCCTT | ATTTGATGTTATTTGTCT | 785 | 41.6 |
| DL4-1 | TGTTTGTTGGTTGAGAAG | AATGAATATGTTACGGGT | 745 | 45.5 |
| YS1-1 | CGAAAAAAAAAGAAGACA | ACCAGGAGAAGAAGGACT | 1265 | 45.0 |
| YS2-1 | AAGATTTTCCCCTTTATT | TTTTAGTTGCATTTGTCG | 557 | 45.2 |
| YS3-1 | TATTAAACATATAGACGC | CAGATTAGAACAACAAAC | 584 | 42.5 |
| YS4-1 | TTGGGTTTTTATTTTTGC | AATTCGGATGTACGCTTA | 635 | 43.9 |
| YS5-1 | TAAAACTTACCAACTTCG | ATACCATTCCTTCACCTA | 719 | 44.2 |
| FJ1-1 | CAGCCATACGCTCTACCT | CGGACTCGAACCAATAAT | 232 | 47.4 |
| FJ1-2 | CGCTCTACCTTTAAGCTA | TTTGGAAATAATCGGACT | 236 | 45.6 |
